# Neuroprotective Activity of *Sibjeondaebo-tang* on A**β** Peptide-Induced Damages

**DOI:** 10.1155/2012/459894

**Published:** 2012-05-15

**Authors:** Hyeon Ju Yim, Jung Hwa Lim, Min Hee Kim, Uk Namgung, Sang Ryong Lee, In Chul Jung

**Affiliations:** ^1^Department of Oriental Medicine, Daejeon University, Daejeon 300-716, Republic of Korea; ^2^Department of Neuropsychiatry, Pusan National University Korean Medical Hospital, Gyeongsangnam-do, Yangsan 626-789, Republic of Korea

## Abstract

*Background*. *Sibjeondaebo-tang* (SJDBT) has been used to treat diverse disorders including neuropsychiatric disabilities in traditional Korean medicine. *Objective*. The present study aims to investigate the potential effects of SJDBT on neuroprotection against A*β* peptide-induced damage using *in vitro* culture and *in vivo* rat brain systems. *Materials and Methods*. PC12 cell viability was analyzed by MTT assay, and neurite arborizations and caspase 3 protein signals in cultured PC12 cells and *in vivo* cortical neurons were analyzed by immunofluorescence staining. Phospho-Erk1/2 protein was analyzed by immunofluorescence staining and western blot analysis. *Results*. In PC12 cells, atrophied cell body and reduced neurite extension by A*β* treatment were recovered by SJDBT treatment. Caspase 3 protein signals were increased in A*β*-treated PC12 cells, but SJDBT treatment decreased apoptotic cell death. Caspase 3 activation in cortical neurons, which was induced similarly by A*β* treatment, was reduced by SJDBT treatment. Furthermore, phospho-Erk1/2 protein levels, which had been decreased by A*β* treatment, were elevated in the cortical neurons by SJDBT treatment. *Conclusion*. These data show that SJDBT may play a role in protecting from damages induced by A*β* in neuronal tissue and further suggest that SJDBT can be explored as the potential therapeutic target for AD treatments in human.

## 1. Introduction

Alzheimer's disease (AD) is the neurodegenerative disease, which is most common in elderly individuals throughout the world, particularly in the developed countries [1]. Extensive studies for the last several decades have made remarkable progress in understanding pathophysiological basis on AD [2, 3]. Accumulation of *β* amyloid (A*β*) peptide and formation of neurofibrillary tangles are the hallmark of AD brain in human. Thus, secretase enzyme that is involved in the processing of A*β* peptide from amyloid precursor protein (APP) is one of the major targets for AD study [4]. A*β* aggregates can alter synaptic transmission, and large aggregates are known to be toxic to neurons [5]. In addition, numerous studies using cultured cells showed that the treatment of A*β* peptide induced cell death even with a low concentration, suggesting that A*β* peptide molecule itself may act as a trigger inducing cell death pathway [6–8]. Thus, A*β* peptide is the major target for mechanistic studies on AD *in vivo* and *in vitro *systems.

*Sibjeondaebo-tang *(SJDBT) is a noted prescription in (DongEuySuSeBoWon) [9], which describes the concept of “*Sasang Constitutional Medicine*”. The basic theory of *Sasang Constitutional Medicine* consists of four types: *Taeyangin*, *Tae-eumin*, *Soyangin*, and *Soeumin*. Each of *Sasang Constitutional* types classified by similar patterns has different characteristics, so there are different patterns of diseases and treatment methods for each type. It was suggested that the proper therapy for *Soeumin, *for instance, is to keep one's body warm and strengthen the blood and *qi*, because their blood and *qi* are weak and stagnant [10]. Lee [9] showed that SJDBT is applicable to treat *Soeumin* exterior diseases, because it might reinforce the healthy *qi* to individuals who are lacking *qi* due to the pathogenic exposure or chronic disease [11]. Consistent with this notion, one recent study has demonstrated that SJDBT might participate in the replenishment of declined energy and cholinergic neurotransmitter synthesis in the mouse cerebral cortex with memory impairment [12].

In the present study, possible protective effects of SJDBT were investigated in cultured PC12 cells and A*β*-injected mouse. Our data indicate that SJDBT is effective to a certain level as a protective agent against A*β* peptide-mediated toxicity in neuronal cells.

## 2. Materials and Methods

### 2.1. Drugs and Chemicals

SJDBT prescription used in the present study is composed of equal amounts of the following herbal ingredients: *Ginseng Radix *(the manufacture's serial number: BH0601, production area: Geumsan-gun, Chungnam, Korea)*, Cynanchi Wilfordii Radix *(the manufacture's serial number: BH0816, production area: Yeongcheon-si, Gyeongbuk, Korea)*, Cinnamomi Cortex Interior *(the manufacture's serial number: SR-0033-10, production area: Vietnam)*, Astragali Radix *(the manufacture's serial number: BH0130, production area: Jecheon-si, Chungbuk, Korea)*, Atractylodis Macrocephalae Rhizoma *(the manufacture's serial number: BH0715, production area: Yeongcheon-si, Gyeongbuk, Korea)*, Angelicae Gigantis Radix *(the manufacture's serial number: BH0615, production area: Pyeongchang-gun, Gangwon, Korea)*, Cnidii Rhizoma *(the manufacture's serial number: BH0801, production area: Yeongyang-gun, Gyeongbuk, Korea)*, Paeoniae Radix Alba *(the manufacture's serial number: BH1030, production area: Jinju-si, Gyeongnam, Korea)*, Citri Pericarpium *(the manufacture's serial number: BH1210, production area: Jeju-do, Korea)*, Glycyrrhizae Radix *(the manufacture's serial number: HPL220C-00608, production area: China)*, Zingiberis Rhizoma Recens *(the manufacture's serial number: BH0910, production area: Muju-gun, Jeonbuk, Korea), and* Zizyphi Fructus *(the manufacture's serial number: BH1205, production area: Gyeongsan-si, Gyeongbuk, Korea).

The drug was obtained from Oriental Medical Hospital of Daejeon University Daejeon, Korea. SJDBT (45 g dry weight) was resuspended in 1 L of water, heat extracted for 3 hrs, and filtered three times. The filtered fluid was distilled using the rotary vacuum evaporator (Büchi 461, Eyela, USA). Concentrated solution was frozen at −70°C for 4 hrs and freeze dried for 24 hrs. The product for SJDBT was 14.7 g, with 29.2% yield from the initial raw materials. The product was kept at 4°C and dissolved in water. The stock solution (10 mg/mL in phosphate buffered saline) was stored at −20°C and used for experiment by diluting with physiological saline solution.

*β*-amyloid peptide was purchased from Sigma-Aldrich (USA), dissolved in H_2_O to 1 mM, and kept at −20°C until use. Galantamine triethiodide (Sigma, USA), a competitive reversible inhibitor of acetylcholinesterase (AChE) was also purchased from Sigma-Aldrich and diluted with H_2_O to concentrations of 0.9 mg/mL and kept at −20°C, freezer until use.

### 2.2. Experimental Animals

Albino mice (7–9-week old, Samtago, Korea) were used in the present study. Animals were placed in an animal room with regulated temperature (22°C), 50% of humidity, and 12-hr-light and 12-hr-dark cycle. They were allowed to eat commercial rat chow (Samyang Co., Korea) and drink water *ad libitum*. All procedures were in strict accordance with the US guidelines (NIH publication no. 85-23 revised in 1985) for the care and use of laboratory animals and approved by the Committee on Use of Live Animals for Teaching and Research at Daejeon University.

### 2.3. PC12 Cell Culture

PC12 cells were cultured in DMEM (GIBCO, USA) supplemented with 5% FBS, 5% horse serum, and 1% penicillin/streptomycin at 37°C incubator supplied with 5% CO_2_. Cells were grown at 75 cm^2^ flask (SPL, Korea) for 3 days and transferred into a new medium. Cells were washed with phosphate buffered saline (PBS) and treated with trypsin-versene solution (Lonza, USA) at 37°C for 5 min for trituration. Detached cells were suspended with DMEM containing 5% FBS and 5% horse serum, plated into a new culture flask with a 1 : 4 dilution, and incubated at a CO_2_ incubator. For immunofluorescence staining experiments, cells (1 × 10^5^ per coverslips) were usually cultured on the coverslips precoated with poly-L-ornithine (0.1 mg/mL, Sigma, USA) and laminin (0.02 mg/mL, Collaborate Research, USA) overnight at room temperature.

### 2.4. MTT Assay

PC12 cell viability was assayed by reduction of MTT [3-(4,5-dimethylthiazole-2-yl)-2,5-diphenyltetrazolium bromide] reagent. Cells (1 × 10^5^/well) were plated in 96-well plate. Cells were treated with 10 *μ*M of A*β* peptide and with different concentrations of 0.3, 0.5, and 1.0 mg/mL of SJDBT for 24 hrs. Then, the cells were treated with MTT solution for 4 hrs, and optical density was measured using spectrophotometer at 570 nm. Cell viability was measured as follows:

(1) $$\begin{array}{cc} & \text{Cell}\;\text{viability}\;\left(\text{\%}\right)\\ & =\frac{\text{optical}\;\text{density}\;\text{of}\;\text{cells}\;\text{treated}\;\text{with}\;\text{drugs}}{\text{optical}\;\text{density}\;\text{of}\;\text{cells}\;\text{treated}\;\text{with}\;\text{saline}\;\text{vehicle}}\times 100. \end{array}$$

### 2.5. Immunofluorescence Staining and Hoechst Nuclear Staining

For immunofluorescence staining, cells or tissues on the coverslips were fixed with 4% paraformaldehyde 4% sucrose in PBS at room temperature for 40 min, permeabilized with 0.5% Nonidet P-40 in PBS, and blocked with 2.5% horse serum and 2.5% bovine serum albumin for 4 hrs at room temperature. Cells on the coverslips were incubated with primary antibody, washed with PBST (PBS plus 0.1% triton X-100) 3 times for 10 min each, incubated with fluorescein-goat anti-mouse (1 : 400 dilution, Molecular probes, USA) or rhodamine-goat anti-rabbit secondary antibodies (Molecular probes) in 2.5% horse serum and 2.5% bovine serum albumin for 1 hr at room temperature, and cover-slipped with gelatin mount medium. For some experimental purpose, Hoechst staining reaction for nuclear visualization was performed between washing steps after secondary antibody reaction. Tissue sections were treated with 25 *μ*g/mL of Hoechst33258 in 0.1% triton X-100 in phosphate-buffered saline solution (PBST) for 10 min. The secondary antibody reaction was performed in a dark place. The merged images were produced by layer blending mode options of the Adobe Photoshop (version 5.5). The primary antibodies were phospho-Erk1/2 kinase antibody (1 : 800, Cell Signaling, USA), cleaved caspase 3 antibody (1 : 500, Cell Signaling), and monoclonal antineurofilament 200 (1 : 400, Sigma-Aldrich).

### 2.6. Western Blot Analysis

Cell or tissue lysates were washed with ice-cold PBS and sonicated under 50–200 *μ*L of triton lysis buffer (20 mM Tris, pH 7.4, 137 mM NaCl, 25 mM *β*-glycerophosphate, pH 7.14, 2 mM sodium pyrophosphate, 2 mM EDTA, 1 mM Na_3_VO_4_, 1% triton X-100, 10% glycerol, 5 *μ*g/mL leupeptin, 5 *μ*g/mL aprotinin, 3 *μ*M benzamidine, 0.5 mM DTT, and 1 mM PMSF). Protein (15 *μ*g) was resolved in 12% SDS polyacrylamide gel and transferred to immobilon polyvinylidene difluoride (PVDF) membranes (Millipore, Bedford, USA). Blots were blocked with 5% nonfat dry milk in PBST (17 mM KH_2_PO_4_, 50 mM Na_2_HPO_4_, 1.5 mM NaCl, pH 7.4, and 0.05% Tween-20) for 1 hr at room temperature and then incubated overnight at 4°C in 0.1% triton X-100 in PBS plus 5% nonfat dry milk containing antibodies. Protein bands were detected using the Amersham ECL kit (Amersham Pharmacia Biotech, Piscataway, USA), with horseradish peroxidase-conjugated secondary goat anti-rabbit or goat anti-mouse antibodies (Transduction Laboratories, Lexington, USA). Relative intensities of the protein bands were analyzed by autoradiography. The antibodies used in the present study were phospho-p44/42 Erk1/2 kinase antibody (1 : 4,000, cell signaling), p44/42 Erk1/2 kinase antibody (1 : 4,000, cell signaling), and cleaved caspase-3 antibody (1 : 1,000, cell signaling).

### 2.7. A*β*-Administered Mouse Model

Albino ICR mice were randomly assigned into (i) normal group, (ii) A*β* peptide-treated control group, (iii) positive control group treated with A*β* peptide plus galantamine, and (iv) experiment group treated with A*β* peptide and SJDBT. 5 *μ*L of A*β* peptide (200 pmol) was microinjected using the glass capillary connected to picoinjector (Harvard Instrument, USA) into the lateral ventricle (0.5 mm posterior to bregma, 1.5 mm lateral to midline, and 2.5 mm ventral to the brain surface) bilaterally. SJDBT extract (400 mg/kg) was orally (Po) administered when A*β* peptide was given, and 8 days after A*β* treatment, galantamine (3 mg/kg) was administered (Ip) on a daily basis for 3 days. The brain was then dissected, and coronal sections (20 *μ*m) were prepared using the cryostat for histological analysis.

### 2.8. Microscopic Analysis

Images from immunofluorescence staining were analyzed by fluorescence microscope (Nikon, Japan), and real-time images of cultured cells were analyzed by phase-contrast microscope. The images were captured and transferred into the computer software (ACT-1). Merged images were analyzed under the image blend mode of the Adobe Photoshop software (version 5.5).

### 2.9. Statistical Analysis

**Figure 1:**
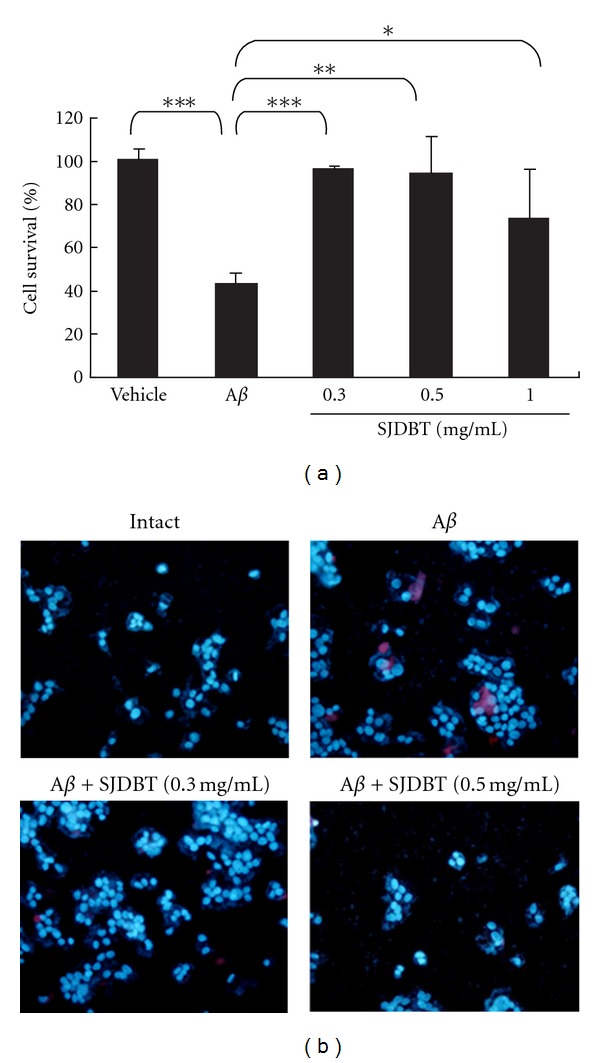
Cell death analysis of PC12 cells. PC12 cells were cultured in the presence of NGF (50 ng/mL) at least for 7 days, and cells were further treated with A*β* in the presence or the absence of SJDBT extract (0.3 mg/mL or 0.5 mg/mL) for 24 hrs. (a) MTT assay. A549 cells were incubated with A*β* (10 *μ*M) alone or in the presence of 0.3–1.0 mg/mL of SJDBT extract. Cells were harvested 24 hrs later for MTT assay. The viability of PC12 cells is the percentage of MTT value relative to normal cells. Mean ± standard error of mean (*n* = 3). *P < 0.05, **P < 0.01, ***P < 0.001 (one-way ANOVA). (b) Immunofluorescence staining of PC12 cells with anticaspase 3 antibody (red) and Hoechst nuclear staining (blue). All the images in (b) are shown as the merged ones of Hoechst nuclear staining and caspase 3 staining in PC12 cells.

Data were presented as mean ± standard error of mean (SEM). A StatView512+ computer software was used for statistical analysis by Student's *t*-test. Statistically significant differences were reported as *P* < 0.05, *P* < 0.01, or *P* < 0.001 (see Figure 1(a)).

## 3. Results

### 3.1. Effects of SJDBT on A*β*-Treated PC12 Cells

To determine possible neuroprotective activity of SJDBT in PC12 cells treated with A*β* peptide, cell survival was measured by MTT assay. The levels of cell survival, when treated with A*β* peptide, were strongly decreased as much as 40% to those of normal group (Figure 1(a)). Cotreatment with SJDBT extract at concentrations of 0.3–0.5 mg/mL enhanced levels of cell survival higher than 80%. However, SJDBT extract at 1.0 mg/mL slightly decreased cell survival. Thus, SJDBT extract at 0.3–0.5 mg/mL was used for the rest of the present study. To determine whether apoptotic signaling pathway was involved in A*β*-induced cell death, caspase 3 activation was measured in PC12 cells. Caspase 3 protein signals, which were not detected in intact PC12 cells, were induced in some of the A*β*-treated cells (Figure 1(b)). It was observed that caspase 3 protein signals were not localized at the central area where the nucleus was identified by Hoechst staining. In cells treated with SJDBT at 0.3 mg/mL, caspase 3 signals were observed in less cells compared to those in A*β*-treated cells. Furthermore, in cells treated with A*β* and 0.5 mg/mL of SJDBT, caspase 3 signals were not detected in any of cultured cells.

**Figure 2:**
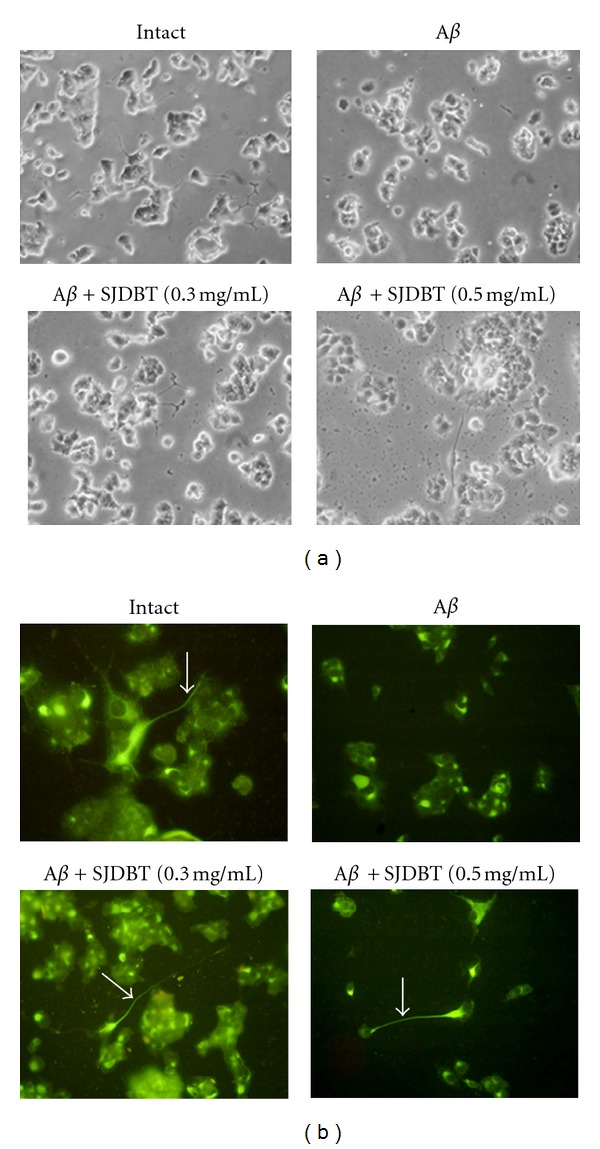
Morphological features of PC12 cells after different treatments. (a) Phase-contrast microscope of PC12 cells. (b) Neurite extension of PC12 cells. Neurite growth processes were visualized by immunofluorescence staining with anti-NF-200 antibodies (green). Neurite extension was marked in arrows in the Figure.

Effects of SJDBT on morphological changes in A*β*-treated PC12 cells were investigated. In NGF-treated PC12 cells, the cell body showed a morphology of spreading and round shapes, and neurite processes were clearly observed (Figure 2(a)). However, cells treated with A*β* revealed shrunken cell body with no clear neurite processes. When the cells were treated with SJDBT extract, neurite growth processes were observed, and particularly longer processes were frequently observed with SJDBT treatment at 0.5 mg/mL. To further examine the pattern of neurite outgrowth, cells were subjected to immunofluorescence staining with anti-NF-200 antibody. While some neurite growth processes were observed in NF-200-stained normal cells, A*β* treatment resulted in overall weak NF-200 staining in PC12 cells with no clear neurite processes. SJDBT treatment at 0.3 mg/mL strongly induced NF-200 staining in cultured cells and showed distinct neurite processes (Figure 2(b)). Longer extension of neurite processes were observed with 0.5 mg/mL of SJDBT extract though overall staining intensity was decreased compared to those treated with 0.3 mg/mL of SJDBT.

### 3.2. Effects of SJDBT on Cortical Neurons Treated with A*β* Peptide

**Figure 3:**
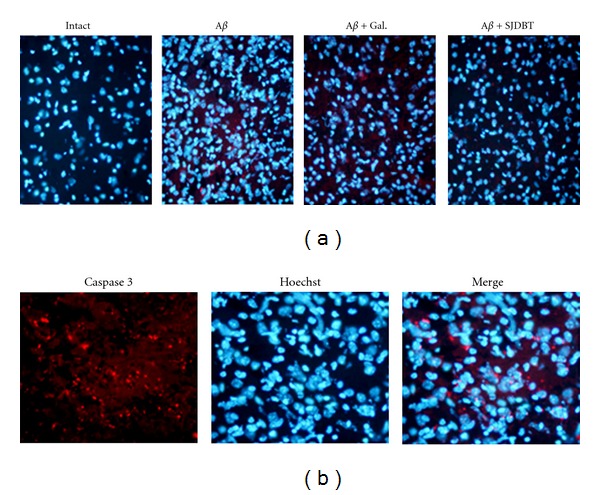
Identification of caspase 3-positive cells in the cerebral cortex. (a) Immunofluorescence view of caspase 3 signals in the cerebral cortex was seen in red. The sections were also stained with Hoechst33258 dye to visualize individual nuclei (blue). (b) Merged image of caspase 3-positive signals with Hoechst-stained nuclei. The image was taken from the brain sections treated with A*β* peptide.

To determine possible neuroprotective effects of SJDBT on A*β*-treated cerebral cortical neurons, levels of caspase 3 protein were analyzed. As shown in Figure 3(a), caspase 3 signals were not detected in normal tissue. Then, caspase 3-positive signals were identified in the brain tissue treated with A*β* peptide though the signals were limited to a few numbers of the cells. Treatment of SJDBT or galantamine as a positive control largely eliminated caspase 3 signals in the tissue (Figure 3(a)). Merged immunofluorescence view of caspase 3 protein signals with Hoechst-stained nuclei revealed that caspase 3 signals were mostly found outside the nuclear area (Figure 3(b)).

**Figure 4:**
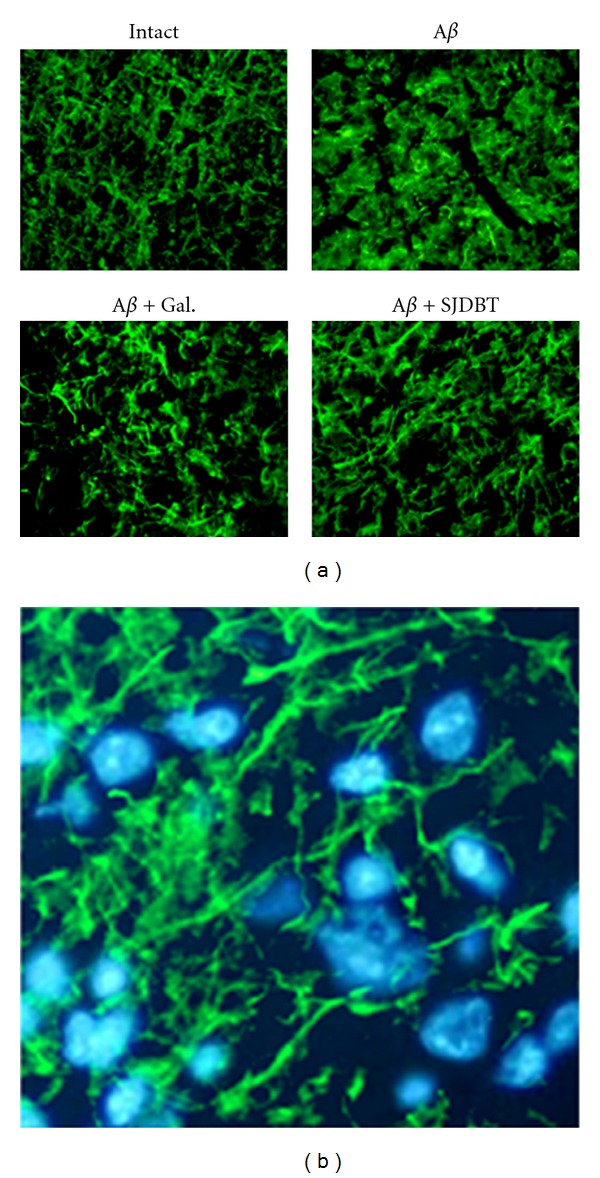
Identification of NF-200-positive cells in the cerebral cortex. (a) Immunofluorescence view of NF-200 signals in the cerebral cortex was seen in green. Notice that NF-200-stained neuronal processes were more intense in intact, galantamine or SJDBT-treated groups compared to A*β*-treated group. (b) Merged view of NF-200-stained processes (green) with Hoechst-stained nuclei (blue). The image is the representative from A*β* and SJDBT-treated group.

Morphological features of cerebral cortical neurons were examined by visualizing neuronal processes with NF-200 immunostaining. It was noted that NF-200 staining intensity in the cerebral cortex was higher in A*β*- and SJDBT-treated groups compared to normal group. Yet, neuritic processes were largely reduced in A*β*-treated group when compared with intact control and galantamine or SJDBT-treated groups. Extended processes were noted particularly in SJDBT-treated group (Figure 4(a)). It was further observed that NF-200-stained neurite processes were labeled distinctively with Hoechst-stained nuclear area (Figure 4(b)).

**Figure 5:**
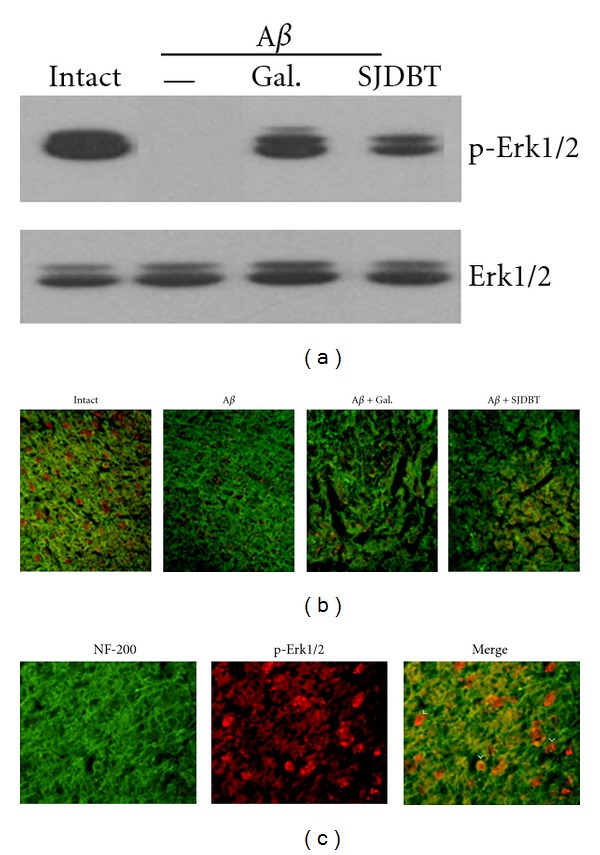
Induction of phospho-Erk1/2 in the cerebral cortex. (a) Western blot analysis. After various treatments into mice, cerebral cortical tissues were dissected out and used for western blot analysis. Western analysis for total Erk1/2 protein for the same transferred membrane was used as an internal loading control. 1: normal, 2: A*β* (200 pmol/5 *μ*L), 3: A*β*+galantamine (3 mg/kg), and 4: A*β*+SJDBT extract (400 mg/kg), (b, c). Immunofluorescence staining of brain sections. (b) Brain sections were used for double immunofluorescence staining for NF-200 protein (green) and phospho-Erk1/2 protein (red), and the merged images were shown in the figure. (c) Immunofluorescence view of phospho-Erk1/2 protein signals (red) in NF-200-stained cortical sections (green). Merged view indicates that the area where phospho-Erk1/2 signals are relatively strong is the central zone surrounded by NF-200-stained processes (arrowheads).

To determine whether SJDBT treatment affects cell survival, phospho-Erk1/2 protein levels, known to be upregulated in diverse cell types showing increased survival activity, were investigated in the cerebral cortex of the rat brain after different treatments. Phospho-Erk1/2 protein was clearly observed in the intact cortical area, but completely abolished after A*β* treatment (Figure 5(a)). Galantamine or SJDBT treatment induced phospho-Erk1/2 protein in the cortical area, although its level was lower than that in the intact tissue. Total Erk1/2 protein maintained at constant levels in cortical tissues after different treatments. Immunofluorescence staining showed that phospho-Erk1/2 protein signals were clearly observed in the intact cerebral tissues. In A*β*-treated cortex, only a weak phospho-Erk1/2 protein signals were observed and increased in the tissues treated with galantamine or SJDBT extract in addition to A*β* (Figure 5(b)). Enlarged view of phospho-Erk1/2 signals overlapped with NF-200 stained image showed distinct subcellular localization; phospho-Erk1/2 signals were not mostly overlapped with those of NF-200-stained processes (Figure 5(c)).

## 4. Discussion

Although several transgenic mouse models of AD have been developed and contributed to understanding AD pathology [13–15], the major obstacles to study AD would be that all of the experimental animals do not display the same pattern of clinical symptoms that occur in AD patients [15]. Neuropathological features such as A*β* peptide deposition in the senile plaque and blood vessels are commonly found in aged primates such as chimpanzees and gorillas, but histological properties including neurofibrillary tangles and neurite atrophy as well as behavioral AD symptoms are not generally observed in these animals as observed in humans [16]. Genetic mouse models in which mutated forms of APP proteins are produced and accumulated in the brain have been developed and widely used to understand at least partial aspects of AD. Several lines of studies showed that A*β* accumulation in the mouse brain was associated with degeneration of the brain tissue and further linked to behavioral learning abnormalities [17–19]. In addition, animals with drugs such as interfering cholinergic neuronal activities have been used for AD phenotypes [20].

Sibjeondaebo-tang (SJDBT), which means a decoction of ten perfect tonifying drugs, was described in (DongEuySuSeBoWon) [9] and is known as one of the major tonifying and replenishing therapies in Oriental medicine. Although there are no records of efficacy or clinical cases, it is considered that the chief virtue of SJDBT is similar to that of Palmigunja-tang (PMGJT) [21, 22], in which the root of SJDBT is used to manic syndrome of “Soeumin” person. Recent studies further provide evidences that SJDBT and its herbal components are effective for treating diverse symptoms. Lee [9] analyzed SJDBT prescriptions for the medical practitioners of several generations and showed that SJDBT is applicable to treat Soeumin's exterior symptoms. Moreover, principal efficacies of herbal components comprising SJDBT have been described elsewhere [23], and there is an increasing number of experimental studies on SJDBT [12, 24–26]; one recent report suggested that SJDBT might participate in the improvement of declined energy production and cholinergic neurotransmitter synthesis in the mouse cerebral cortex with memory loss [12], which is consistent with classical hypothesis on SJDBT efficacy. Another study suggested that SJDBT might protect the spontaneous and glutamate-induced neuronal damages in cultured cerebrocortical cells of mouse [26].

Based on previous studies and classical descriptions, we hypothesized that SJDBT might play a protective role for neuronal cells *in vitro* as well as mouse brain *in vivo*. As the first step to determine whether A*β* peptide has any toxic effect on cultured PC12 cells, A*β*-treated PC12 cells were examined by MTT assay, morphology assay, and survival assay. MTT assay showed clear decreases in cell viability. Furthermore, investigation of cell morphology by Hoechst-stained nucleus and phase-contrast microscopic analysis showed shrunken cell body shape. A*β* treatment induced caspase 3 protein signals in some PC12 cells, suggesting that A*β* treatment might activate both apoptosis and necrotic cell death pathways, as it has been implicated before [8, 27]. Besides the activation of cell death machinery, A*β* caused decreased neurite extension in NGF-treated PC12 cells.

When A*β*-treated PC12 cells were cotreated with 0.3 mg/mL or 0.5 mg/mL of SJDBT extract, a few changes were noted. Cell viability as determined by MTT assay and a pattern of neurite extension were improved to the levels similar to normal cells. Then, SJDBT treatment reduced the number of caspase 3-positive cells. However, it should be noted that caspase 3-positive cells by A*β* treatment were very limited, while cell death profile as determined by MTT assay was much more noticeable. This implicates that both apoptotic and necrotic death pathways are involved in A*β*-mediated cell death, but the necrotic pathway may play a major role in cell toxicity in PC12 cells. Based on the protective effects of SJDBT on A*β*-treated PC12 cells, similar effects by SJDBT extract were further examined in cerebral cortical neurons in the rats. A*β* injection into the lateral ventricle caused increased signals of caspase 3-positive cells in the cerebral cortex, although caspase 3-positive signals were limited to only a few numbers of cells. Caspase 3-positive cells were scattered throughout the cortical area, implicating homogeneity in A*β* toxicity to cortical neurons. Oral administration of SJDBT extract into the mice caused decreased levels of caspase 3-positive cells in the cerebral cortex, suggesting the possible inhibitory activity of SJDBT on apoptosis.

When the morphological features of A*β*-treated individual cells were compared between cultured PC12 cells and *in vivo* cortical neurons, cytotoxicity appears to be much more severe to PC12 cells than *in vivo* neurons. For instance, NF-200-stained neurons or Hoechst-stained nuclei *in vivo* did not show any distinctive differences among brain tissue after different treatments, whereas the shrinkage of PC12 cell bodies was generally observed after A*β* treatment. Despite mild morphological effects of A*β* peptide on brain tissue *in vivo*, A*β*-induced changes in molecular targets in relation to cell survival or death were observed. Levels of phospho-Erk1/2 protein, a signal for survival [28], were remarkably decreased by A*β* treatment in cortical region and recovered by galantamine or SJDBT treatment. Immunofluorescence staining analysis showed that phospho-Erk1/2 signals were largely localized in the cell body region, particularly at the nuclear area. Since one of the major downstream targets of phospho-Erk1/2 activity is the transcription factors such as cAMP responsive element binding protein (CREB), phospho-Erk1/2 could function mostly in the nucleus besides cytoplasm.

## 5. Conclusions

Our experimental study provided evidence that SJDBT extract can regulate molecular targets in the cell and induce neuroprotective pathways *in vivo* as well as *in vitro* systems. Since SJDBT is a mixture of several herbal ingredients, it is reasonable to state that diverse chemicals comprising SJDBT might interact with numerous cellular targets given A*β*-induced toxicity. It should be also mentioned that while SJDBT appears to protect neuronal cells, whether SJDBT improves behavioral abnormality in association with AD pathology remains to be explored. It is critical to develop more convincing animal model representing neurological characteristic of AD. By combining diverse principles and technical applications, more specific and precise examinations on the efficacy of SJDBT would be possible.
